# Correlations Between Department and Training Program Online Presence and Women in Orthopedic Surgery Training

**DOI:** 10.1089/whr.2022.0081

**Published:** 2023-03-01

**Authors:** Sarah Adkins, Dorothy Hughes, Mary Zimmerman, Kimberly Templeton

**Affiliations:** ^1^ University of Kansas School of Medicine, Kansas City, Kansas, USA.; ^2^ University of Kansas School of Medicine-Salina, Population Health and Surgery, Salina, Kansas, USA.; ^3^ Department of Population Health, University of Kansas School of Medicine, Kansas City, Kansas, USA.; ^4^ Department of Sociology, University of Kansas-Lawrence, Lawrence, Kansas, USA.; ^5^ Department of Orthopaedic Surgery, University of Kansas Medical Center, Kansas City, Kansas, USA.

**Keywords:** orthopedic surgery, websites, social media, women, gender, residents

## Abstract

**Background:**

Orthopedic residency programs increasingly use websites and social media to reach students. This accelerated during the COVID-19 pandemic, especially as away rotations became limited. Women remain a minority of orthopedic residents, and there are no data that indicate the correlation between department/program website content or social media presence on the gender diversity of residency classes.

**Methods:**

Orthopedic department websites were assessed between June 2021 and January 2022 to identify program director's gender, as well as the gender composition of the faculty and residents. Instagram presence for the department and/or program was also identified.

**Results:**

There was no correlation found between the residency program director's gender and the gender diversity of residents in a given program. The percentage of women faculty identified on a department website was significantly correlated with the percentage of women residents in the program, regardless of the program director's gender. While there was an increase in the percentage of women residents among programs with Instagram accounts for the class that started in 2021, this was negated when the percentage of women faculty was taken into account.

**Conclusion:**

Efforts on multiple fronts will be needed to increase the number and percentage of women applying for and training in orthopedic surgery. Given the increasing use of digital media, we need a better understanding of what information, including faculty gender diversity, can be conveyed through this format that is useful for women medical students interested in orthopedic surgery to address their concerns about the field.

## Introduction

Women represent a small minority of academic orthopedic faculty^1^ and residents.^1,2^ The latter has not changed significantly over time and is increasing at a rate slower than any other surgical subspecialty that is composed primarily of men.^1^ While women in orthopedic surgery continue to face issues such as microaggressions during their training,^3^ the view of the entire field as sexist is part of the “hidden curriculum” and one of many factors that can deter women from pursuing a career in orthopedic surgery.^4^ While women and men medical students have been found to have similar perceptions of orthopedic surgery, women are more likely to see the field as “male dominated.”^5^ These and other factors, such as lack of strong mentors in the field and limited exposure to musculoskeletal topics in medical schools,^6^ have led to women representing only 16%–20% of applicants to the orthopedic surgery residency programs.^7^

Medical students have faced significant unique challenges in the residency application process during the COVID-19 pandemic, in part, related to the discontinuation of most away rotations, as recommended by The Coalition for Physician Accountability. Before the pandemic, medical students interested in orthopedic surgery apply to an average of 75 programs,^8^ leading them to rely on information about the majority of programs to which they apply from sources other than through in-person interactions. Women medical students who participate in in-person clerkships have been found to be less likely to see the field as sexist after than they did before that experience.^9^ Thus, women medical students interested in orthopedic surgery, could have been negatively impacted by the lack of away rotations and not having the ability to interact with a variety of residents and orthopedic surgeons.

Orthopedic departments and residency training programs have been in the positions of determining how best to inform students about their programs and to encourage them to apply; this was exacerbated by the pandemic. One approach has been an increased use of websites and social media.^10^ The use of Instagram, in particular, has accelerated during the COVID-19 pandemic.^10^ Bram et al.^11^ found that 118 programs had Instagram accounts, a higher number than accounts on any other social media platform.

Of these, 102 were created during the COVID-19 pandemic. Common topics of social media posts include spotlights of people in the program and comments about the residency program in general.^11^ Abbas et al.^12^ found Instagram posts from orthopedic residency programs most commonly included information specific to their training programs and social photographs, with fewer posts containing educational, clinical, or research content. The total number of posts and the numbers of educational, surgical, social, and program information posts had significant correlations with increased follower count. However, few social media posts were found to mention women in orthopedic surgery.^11^

Websites and social media posts are typically the first interaction an interested student has with a program. These can not only provide information of interest to all applicants but can also address factors such as the presence of female and minority faculty and program reputation for diversity, which have been found to be more important to women applicants.^13^ A prior study indicated programs with more women faculty and women in leadership positions (broadly defined as section chief, residency program director, or fellowship program director) noted on their websites had more women residents in their training programs.^14^

However, the information obtained from websites was confirmed by querying program residency coordinators. There is no study that focuses only on information that is readily available to students on a department website in terms of the number of women faculty or on the presence of a woman residency program director (rather than the more broadly defined program leadership), and the correlation of those factors with the percentage of women orthopedic residents. There is also no indication of whether having a social media presence impacts the gender diversity of orthopedic residents within a program.

## Methods

Between June 2021 and January 2022, we accessed and assembled data provided by orthopedic surgery residency programs across the United States on public websites regarding the perceived gender of program directors, resident classes, and faculty. We excluded programs that did not have publicly accessible resident classes, faculty rosters, or both. There was no information available on the websites that would help to identify the presence of transgender faculty or residents. We counted total faculty as well as the subtotal of faculty listed as MD/DO/PhD; it was important to exclude those in other health professions, who may have been department faculty, but would bring different perspectives than orthopedic physicians and surgeons or orthopedic surgeon-scientists.

In addition, we create a binary variable for Instagram presence (programs with an Instagram account = 1 and those without = 0). Our outcome variable was proportion of women residents (in programs). First, we described all variables using measures of central tendency to determine trends in frequencies and percentages of women over time. Our independent variables were the use of Instagram and the proportion of women program directors and faculty. Gender was presumed by the authors based on names and pictures on program websites. Programs in which this could not be discerned were not included in the analysis.

We used Wilcoxon Rank-Sum tests to determine whether the proportion of women residents was meaningfully different among programs with and without Instagram accounts. Using simple linear regression, we examined the associations between our independent variables and our dependent (outcome) variable, proportion of women residents. There were too few women chairs to allow for valid statistical tests among programs with and without women chairs, so we did not include this in our analysis. As this study used publicly available data, this secondary data analysis did not require IRB approval. All analysis was conducted using Stata (StataCorp LLC, College Station, TX).

## Results

After evaluating all 204 orthopedic residency programs in the United States, 60 were excluded due to missing data, bringing the number of programs in our study to 144. Among these, we found 5377 total MD/DO/PhD credentialed faculty, of which 722 (13%) were women. Across resident classes, the total population was 3359, of which 596 (18%) were women. Women represented a minimum of 3.57% and a maximum of 35% of residents in programs, with a median of 16.67% and a mean of 17%.

**FIG. 1.**
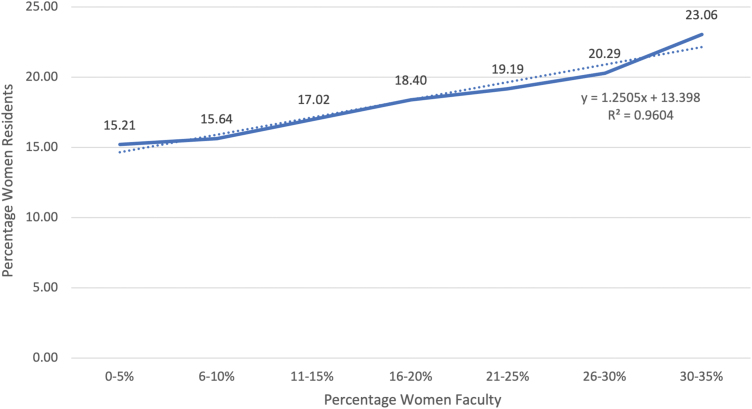
Percentage of women residents associated with percentage of women faculty across orthopedic surgery residency programs.

Analysis of programs as a group indicated that as the percentage of women on faculty increased, the percentage of women residents increased as well. Overall, there was a significant positive relationship between the percentage of female faculty with MD/DO/PhD credentials and the percentage of female residents at a given program (*p* = 0.003), regardless of the gender of the program director (Fig. 1). In a simple linear regression, the beta coefficient was positive, suggesting that as the percentage of women faculty increased by one percentage point, the percentage of women residents increased by 0.215% points.

There were 14 women and 130 men program directors identified on program websites. The percentage of women residents in a program with woman program director was 14.2% and that for programs with a man program director was 17.6%. There was no statistically significant difference between percentages of women residents in programs with women program directors and those with men program directors.

**FIG. 2.**
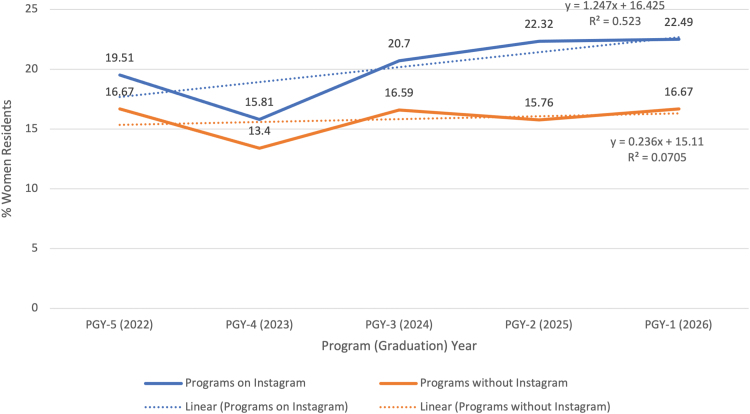
Percent of women orthopedic surgery residents by program year correlated with program Instagram presence.

One hundred programs were identified that had Instagram accounts. These programs accounted for 2443 total residents, of which 449 (18%) were women, and of 4,045 MD/DO/PhD faculty members, 570 (14%) were women. Using the Wilcoxon rank-sum test, programs with an Instagram presence had significantly larger percentages of women in their program (*p* = 0.023). Women represented 16.7% of residents in programs without Instagram accounts and 22.5% of residents in programs with Instagram accounts. When tested by program year, there was no statistically significant difference in percentage of women residents between programs with or without Instagram accounts for those in the PGY-5 year (*p* = 0.14), PGY-4 year (*p* = 0.22), or PGY-3 year (*p* = 0.52). For those in the PGY-2 year, this was close to statistical significance (*p* = 0.05) and was significant for those in the PGY-1 year (*p* = 0.02), that is for those who started training in 2021 (Fig. 2).

In a simple linear regression, the beta coefficient for the presence of an Instagram account was 3.17, suggesting that Instagram had a positive effect directionally, and if a program moved from no Instagram to having an Instagram presence, we would expect to see a 3.17% point increase in the percentage of women residents. However, when we controlled for the number of women faculty in the regression, the presence of an Instagram account was no longer significant (*p* = 0.330). In addition, there was no significant difference between the current first-year trainees and those in other years in training after accounting for the number of women faculty. Taken together, Instagram and percent women faculty together explain about 7.86% of variation in the percentage of women residents among programs.

## Discussion

Women continue to represent a small minority of orthopedic surgeons in practice and in training, primarily due to low rates of residency applications. While the percentage of women applicants is increasing, it is still the lowest among surgical specialties.^15^ A variety of factors have been identified that may dissuade women from choosing orthopedic surgery as a career, including a lack of mentoring, negative personal experiences,^6^ and the “hidden curriculum.”^4^

While there is increasing reliance on on-line information and programming to address these issues and encourage women to consider orthopedic surgery as a career, conveying the culture of a specific training program can be difficult through virtual platforms and interactions. A culture that is welcoming to and supportive of women could, in part, be demonstrated by the presence of greater numbers of women faculty and a woman program director in program on-line materials. Greater gender diversity among faculty has been found to be more important to women than men applicants to residency programs, both in orthopedics^13^ as well as other surgical specialties.^16^

This study found that programs with greater numbers of women faculty also had higher percentages of women residents. While the relationship between faculty and resident gender has previously been demonstrated,^14,17^ the current study specifically looked at the total number of women faculty in a program, including orthopedic surgeons and researchers, who were identified on a department or program website. This reflects the information readily available to students, rather than a comparison with the number of women faculty listed in the Association of American Medical Colleges (AAMC) database,^17^ relying on calling programs for confirmation of faculty gender,^14^ or other sources of information to which students do not have access.

Having more women faculty, both clinicians and researchers, may be indicative to prospective applicants of the culture of the department and/or the availability of more gender-concordant mentors. Luc et al.^18^ found that women surgeons and surgical trainees were more likely than men to feel that mentorship played an important role in their career paths, but were less likely to have access to an institutional mentoring program and were also more likely than men to seek gender-concordant mentoring.

This study found that increasing the percentage of women faculty by one percentage point is associated with an increase in percent women residents of 0.215% points. This is less than what has been demonstrated previously in other areas of medicine: in an assessment of the 20 largest specialties, Chapman et al.^17^ found that, for each percentage increase in women faculty, the percentage of women trainees increased by 1.45%. While no specific percentage of women faculty target is recommended, these data indicate that one intervention to increase the number and percentage of women training in orthopedic surgery could be to focus on recruiting more graduates into becoming academic surgeons. In addition, women in academic departments need to be mentioned on department or training program websites for those outside of the department, including students, to be aware of their presence. Kuhns et al.^19^ found a statistically significant difference between the number and the percentage of women faculty found on academic orthopedic department websites (10%) and their representation in the AAMC faculty database (19%).

Women represented 10% of program directors in this study, with no apparent correlation between program director gender and gender composition of resident classes. The results are similar to what has been described in other areas of surgery, including plastic surgery^20^ and general surgery,^21^ in which program director gender did not influence the gender composition of training programs. However, this finding does not confirm what has been published regarding the 10 largest specialties, based on enrollment, in which the presence of a woman program director correlated with significantly higher percentages of women trainees.^22^ In addition, the results of this study also differ from what has been reported in the orthopedic literature regarding the relationship between women in leadership roles and women in training: when “leadership” is broadly defined, including program directors and others, a significant relationship has been reported.^14^

However, this study is the first to look specifically at the relationship between residency program director gender and proportion of women in orthopedic training, and no similar relationship was found. Assessing the relationship between program director and trainee gender is important, given the significant role program directors play in training programs. In addition to their roles in overseeing clinical education and resident selection, program directors have been described as “the most important and central figures in the training of orthopedic surgery residents,” who “must advocate for residents and their education on multiple levels.”^23^ Program directors are also typically involved in outreach to students. Having women in these positions can indicate to women students that they would have an advocate and mentor at that program and can also indicate a culture that is welcoming to women in leadership positions.

This study found that the mere presence of an Instagram account had no impact on the percentage of women residents within the program, comparing those who would have applied during the COVID-19 pandemic, a time of increased use of social media by residency programs, to residency classes that previously matriculated. The use of social media among orthopedic training programs to engage and recruit students is a relatively new phenomenon.^24^ There are limited data to indicate the impact of social media on medical education or residency training programs, with most such research being published before the COVID-19 pandemic. In 2012, in a study of osteopathic medical students and trainees, Schweitzer et al.^25^ found that about one-fourth of students obtained information about training programs through social media platforms.

A systematic review published in 2017 noted that departments were starting to use social media as part of the resident recruitment process.^26^ In light of increasing rates of use of social media among current medical students, including to find information about training programs,^27^ greater program presence on social media,^28^ and issues with relatively limited clinical experiences, especially during the COVID-19 pandemic, the influence of websites and a social media presence on generating interest in the specialty and in specific training programs has likely increased and may become a more important factor than what was noted in the past.^29^ However, this study did not assess the content of Instagram posts. While Bram et al.^11^ noted that a small percentage (12.3%) of orthopedic program social media posts mentioned women orthopedic surgeons, they gave no further detail or context. Social media has been identified as a way for women surgeons and surgical trainees to identify gender-concordant mentors, given the relative dearth of women in the field.^18^ With the important role of social media in current and likely future generations of medical students, it will be important to better understand what is being posted, if this is sufficient to address views regarding gender bias in orthopedic surgery, and identify ways to leverage this medium as another way to recruit women into orthopedic surgery.

### Limitations

Limitations of this study include that it was descriptive in nature, using a convenience sample of residency training programs that had websites with complete information. The study results indicated associations, but are not to be construed as causations. In addition, it assessed gender of faculty and program directors through review of department and program websites without confirmation through the AAMC database or by contacting programs.

However, the intent was to mimic a search that any medical student might perform as they are considering residency programs. In addition, the number of women program directors may have been too small to identify significant differences in residency program composition and will need to be reexamined as more women assume these roles. This study also assessed faculty and program directors through a binary gender lens. Transgender or other nonbinary gender is not noted on websites and is a limitation of both the study and currently available information. Hopefully, with the increased awareness and work on this issue, this information will be more accessible and the issues of transgender faculty and residents in orthopedic surgery discussed, to allow students to see that the field is welcoming to all. In addition, we looked for the presence of a department or residency program on Instagram.

Other social media sites, such as Facebook or Twitter, could also be evaluated. However, Instagram is the platform that has seen the greatest increase in the presence by orthopedic departments and use by students, especially during the pandemic. We also only looked at the presence of an Instagram account, not the number, frequency, or content of posts. The last, in particular, is significant moving forward. As a first step, we need to better understand what information could be of interest to potential women applicants in the field, especially to address concerns about whether the culture of orthopedic surgery, or that of specific programs, is welcoming to and supportive of women.

It is important to note in this context that social media content could be discouraging as well as encouraging to potential women applicants. We then need more clarity on what is being posted on residency program social media accounts, whether this information promotes the careers and accomplishments of women in orthopedic surgery, and if it provides the information that is of interest to women students.

## Conclusion

Increasing the number and percentage of women applicants and residents in orthopedic surgery will require interventions from multiple levels. Although we have returned to in-person interactions postpandemic, academic departments and residency training programs will likely continue to use their website and social media platforms to provide information to prospective orthopedic surgery applicants about the activities and culture of a program, as students only have the opportunity for in-person experiences at a minority of programs in which they are interested.

Social media has the potential to address questions about resident quality of life, happiness, and camaraderie, all of which have been found to be important to potential applicants.^29^ However, understanding the culture of orthopedic surgery, especially in terms of how welcoming it is to women and in addressing perceptions of the field, could be difficult over a virtual format, and while having an on-line and social media presence and a woman program director are likely important, they are not sufficient in isolation to attract women into orthopedic training programs. As a primary driver to increasing the proportion of women residents is a greater number and percentage of women faculty, we need to continue to encourage women orthopedic surgeons to pursue careers in academia, support and promote them to leadership positions, and then make them and their work visible on department websites.

We also need to know more from women students about what aspects of websites and social media postings are of interest to them, which could help residency programs better leverage these media to communicate that orthopedic surgery is a viable career for women and to address their concerns, especially about the “hidden curriculum” or gender biases in the field and to demonstrate the diversity in the field and in a given program.^30^ By taking a multipronged approach, including these and other interventions, we can hopefully start to increase the number of women applicants and trainees and their overall proportion in orthopedic surgery.

## Abbreviation Used

KU: University of Kansas
